# Intravenous Immunoglobulin in Kawasaki Disease—Evolution and Pathogenic Mechanisms

**DOI:** 10.3390/diagnostics13142338

**Published:** 2023-07-11

**Authors:** Pallavi L. Nadig, Vibhu Joshi, Rakesh Kumar Pilania, Rajni Kumrah, Jayakanthan Kabeerdoss, Saniya Sharma, Deepti Suri, Amit Rawat, Surjit Singh

**Affiliations:** 1 Pediatric Allergy Immunology Unit, Department of Pediatrics, Postgraduate Institute of Medical Education & Research, Chandigarh 160012, India; poorvinadig91@gmail.com (P.L.N.); vibhujoshi2411@gmail.com (V.J.); kumrah.rajni@yahoo.com (R.K.); drsaniya.sharma@gmail.com (S.S.); surideepti@gmail.com (D.S.); rawatamit@yahoo.com (A.R.); surjitsinghpgi@rediffmail.com (S.S.); 2 Pediatric Biochemistry Unit, Department of Pediatrics, Postgraduate Institute of Medical Education & Research, Chandigarh 160012, India; jayakanthankk@gmail.com

**Keywords:** Kawasaki disease, treatment, intravenous immunoglobulin, pathogenesis, coronary artery abnormalities, innate immunity, adaptive immunity

## Abstract

Kawasaki disease (KD) is an acute vasculitis of childhood that affects the medium vessels with a special predilection to the involvement of coronary arteries. The major morbidity of this disease is due to coronary artery aneurysm, which occurs in about 25–30% of untreated cases. For decades now, intravenous immunoglobulin (IVIg) has consistently been shown to reduce the risk of CAAs to less than 5%. However, the mechanism of immunomodulation remains unclear. Several studies on the role of IVIg in the modulation of toll-like receptor pathways, autophagy, and apoptosis of the mononuclear phagocytic system, neutrophil extracellular trap, and dendritic cell modulation suggest a modulatory effect on the innate immune system. Similarly, certain studies have shown its effect on T-cell differentiation, cytokine release, and regulatory T-cell function. In this review, we discuss the potential mechanisms underlying the immunomodulatory actions of IVIg in patients with Kawasaki disease. Furthermore, we provide a summary of the evidence regarding various infusion protocols and dosages utilized in the treatment of KD patients.

## 1. Background

Kawasaki disease (KD) is the commonest medium vessel vasculitis in children. KD is one of the commonest causes of acquired heart disease in children in developed countries. The most critical complication of KD is the development of coronary artery abnormalities (CAAs) [1]. The incidence of CAA is observed up to 25% in untreated patients, effectively reduced to <5% with timely treatment. Intravenous immunoglobulin (IVIg) remains the standard of care in all children with KD. Approximately 10–20% of patients with KD remain refractory to IVIg and require additional therapy. In this manuscript, we review the evolution and pathogenic mechanisms of IVIg therapy in KD.

## 2. History of the Evolution of IVIg as a Treatment for KD

After the initial description of the disease in 1957 by Dr. Tomisaku Kawasaki, aspirin and prednisolone were used extensively for treating KD [2]. In 1979, Kato et al. showed that aspirin reduced the incidence of CAAs (11%); however, the use of steroids was associated with increased chances of CAAs (64.7%) in KD [3]. Following this study, the usage of corticosteroids in KD halted, which paved the way for exploring newer options. One such option was IVIg.

Furusho et al. [4], in 1984, carried out a landmark study on the use of IVIg in patients with KD [5]. They performed a multicentric controlled trial of IVIg with aspirin versus aspirin alone. The control group received aspirin at a dose of 10–30 mg/kg/day for 3 months, while the treatment group received IVIg at a dose of 400 mg/kg/day for 5 days within 7 days of onset of illness. The study results showed that none of the patients in the IVIg group developed CAAs during the follow-up period, while 17% of the patients in the control group developed CAAs. Additionally, the IVIg group experienced early resolution of fever compared to the control group. These were significant findings because they first time demonstrated the efficacy of IVIg in preventing the development of CAAs in KD [5]. As a result of this study, IVIg became an important component of therapy in KD. Following this landmark study, several other studies were conducted by different authors to further establish the optimal dosage and duration of IVIg treatment in patients with KD. These subsequent studies aimed to refine the use of IVIg, to maximize its benefits and minimize potential side effects. Overall, the study by Furusho et al. and subsequent research on IVIg in KD have improved the management and outcomes of patients with this condition [6,7,8].

Further, Furusho et al. and Ogawa et al. investigated the efficacy of different doses of IVIg in the KD [9,10]. They compared the outcomes of patients who received a dose of <1 g/kg of IVIg to those who received a higher dose (>1 g/kg). Both studies reported that the low dose of IVIg (<1 g/kg) was ineffective in preventing the development of CAAs in KD. In contrast, the higher dose of IVIg was found to be more effective in reducing the risk of CAAs. 

In 1990, a randomized controlled trial involving 105 Japanese children with KD considered to be at high risk of developing CAAs was randomized between 1 g/kg single dose and 2 g/kg single dose, demonstrating higher efficacy with the latter [11]. Results of this study showed that a higher dose (2 g/kg) was more effective in preventing the development of CAAs than those who received 1 g/kg. Later in 1991, a large multicentric randomized control trial in the United States by Newburger et al. compared the efficacy of daily infusions of 400 mg/kg/day for 4 consecutive days versus IVIg as a single 2 g/kg infusion over 10 h [12]. The study showed that children treated with a single high dose (2 g/kg) of IVIg infusion had several advantages over four consecutive daily infusions protocol. There was early defervescence with a shorter mean duration of fever in the single high dose infusion group—29.3% of children were febrile on day 3 in four consecutive day infusions group compared to 19.1% in the single infusion group; *p* = 0.028. Further, the single high-dose infusion group had lower systemic inflammation in the form of lower C-reactive protein (CRP) (*p* = 0.017), higher serum albumin (*p* = 0.002), and lower alpha-1 antitrypsin (*p* = 0.007) at 2 weeks of follow-up. More importantly, the single high-dose infusion group had a lower prevalence of CAAs when compared to the four-day infusion group (4.6% vs. 9.1% at 2 weeks follow-up, *p*= 0.042; 3.9% vs. 7.2% at 7 weeks follow-up, *p* = 0.098). Among the 4-day infusion group, it was noted that lower IgG levels on day 4 were associated with a higher prevalence of CAAs (*p* = 0.002) and a greater degree of systemic inflammation [12]. The mechanisms underlying the effectiveness of a single large dose of IVIg in KD treatment are not fully understood. However, two possible explanations were suggested by Newburger et al.; one possibility is the neutralization of superantigens that bind nonspecifically to receptors on antigen-presenting cells. Another possibility is the binding of gamma globulin to FcγR1, leading to the rapid downregulation of the cytokine storm. Nonetheless, a single high dose of IVIg emerged as a mainstream treatment following this study. 

## 3. Mechanisms of Action of IVIg

The study conducted by JC Burns and colleagues investigated the effect of high doses of IVIg on natural killer (NK) cell activity in peripheral blood in patients with KD [13]. The authors demonstrated that IVIg could inhibit the interaction between NK cells and endothelial cells, suggesting a potential mechanism for its therapeutic effects in KD [13]. Mouthon L et al. in 1995 provided an elaborate description of the mechanisms of action of IVIg in immune-mediated disorders [14]. High-dose IVIg has been used in various autoimmune and inflammatory disorders, and it is hypothesized to exert its immunomodulatory role in both Fab-dependent and Fab-independent manners. The immunomodulatory effects of IVIg involve interactions with different components of the immune and vascular systems, leading to the downregulation of inflammation. IVIg can target various cell types, including endothelial cells and cells involved in both innate and adaptive immunity [14]. In the following discussion, we summarize the possible mechanisms of the immunomodulatory role of high-dose IVIg. Proposed mechanisms involve the provision of the anti-idiotypic antibodies, clearance of autoantibodies by binding with Fc receptors, blockade of adhesion molecules, and activation of the inhibitory Fc receptor (i.e., FcγRIIB) on macrophages. IVIg has the potential to neutralize the cytokines and superantigens as well as augment the T-cell suppressor activity.

### 3.1. Effect of IVIg on the Innate Immune System (Figure 1)

The innate immune system comprises immune cells such as neutrophils, monocytes, macrophages, dendritic cells, natural killer cells, and natural killer-T cells; physical barriers such as epithelial cells and endothelial cells. They act through several pattern recognition receptors such as toll-like receptors (TLRs), nucleotide oligomerization domain (NOD)-like receptors, and cytokine receptors, through which downstream pathways induce secretion of chemokines, activation of inflammasomes and recruitment of innate immune cells [15,16]. It has been observed that the innate immune system is actively involved in inflammation in the acute phase of KD as observed by predominant neutrophilic leucocytosis, and monocytosis, elevated levels of the damage-associated molecular pattern (DAMPs) such as high mobility group box 1 (HMB1) and S100 proteins in patients with KD. Cellular infiltrates in the coronary artery during the acute stage are composed of macrophages and neutrophils. Coronary artery inflammation could be induced by pathogen-associated molecular patterns (PAMPs) in mouse models [17].

**Figure 1:**
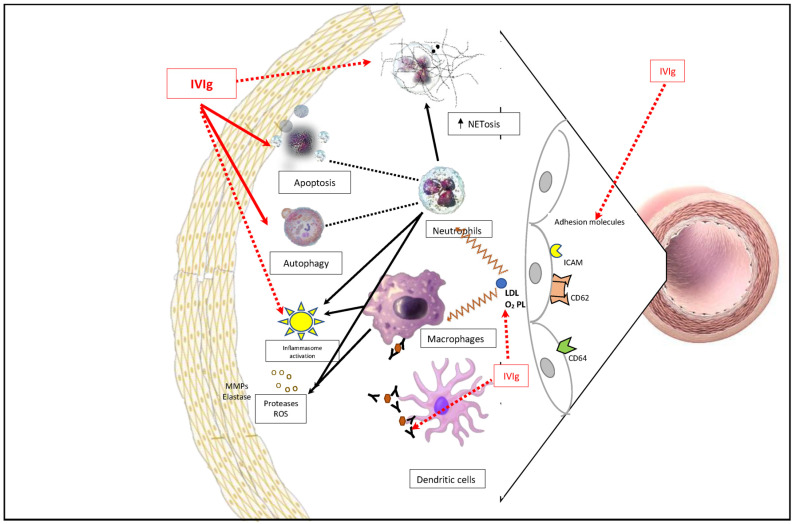
Effect of IVIg on innate immune cells-neutrophils/macrophage and dendritic cells: Expression of adhesion molecules such as CD62, CD64, and ICAM is increased in Kawasaki disease, which helps in the translocation of immune cells into the intimal layer of the vessel wall. PAMPs/DAMPs such as LDL and oxidized phospholipids activate the phagocytic cells. The possible immunomodulatory action of IVIg is indicated by the solid arrow (activation) and dotted arrow (inhibition). ICAM: Intracellular adhesion molecule; PAMPs: pathogen-associated molecular patterns; DAMPs: damage-associated molecular pattern; LDL: low-density lipoprotein.

#### 3.1.1. Interaction with Neutrophils

Neutrophilic leukocytosis is seen in most children with KD during the acute phase, morphologically characterized by toxic granulations and cytotoxic vacuolations, which tends to be higher in KD patients with concomitant CAAs [18]. Takahashi et al. demonstrated the infiltration of numerous neutrophils (anti-elastase-positive neutrophils) in CAAs during the early phase of KD (Days 7–9 of illness) before the infiltration of macrophages and lymphocytes, suggesting that neutrophils are involved in the initial damage that occurs to the coronary arteries [19]. Neutrophils are constitutively programmed to undergo apoptosis, thus reducing the inflammatory process. However, defective apoptosis has been demonstrated in patients with KD during the acute phase [20,21]. Takeshita et al. demonstrated that IVIg promoted neutrophil apoptosis in the in vitro culture of lipopolysaccharide-stimulated neutrophils (which represents the activated neutrophils in vivo) in a dose-dependent manner, and no such observations were made on unstimulated neutrophils [22]. Tsujimoto et al. have also observed similar findings in another in vitro study [23]. These studies suggested the possible mechanism of induction of neutrophil apoptosis by high-dose IVIg in patients with KD.

Autophagy, a cellular process involved in the degradation and recycling of damaged or unnecessary cellular components, has been implicated in limiting neutrophilic inflammation [15]. Impairment of autophagy has been observed in KD, and IVIg therapy has been found to have some impact on autophagy markers. Huang et al. [24] showed that mRNA expression of autophagy markers such as LC3, Beclin 1, and ATG16L1 was lower in the leukocytes of patients with KD compared to the control groups (febrile and healthy control groups), suggesting impaired autophagy in KD. Furthermore, investigators also evaluated the impact of IVIg therapy on autophagy markers and showed that the expression of these markers significantly increased 21 days after IVIg therapy [24]. This finding suggests that IVIg treatment may have a restorative effect on autophagy in patients with KD and could contribute to the regulation of neutrophilic inflammation and help resolve the inflammatory response associated with KD.

The role of neutrophil extracellular trap formation (NETosis) has also been investigated in KD (Figure 1). Yamashita et al. investigated the stimulation of NET formation by sera of patients with KD in the acute phase (pre-IVIg) and during the convalescent phase (5–10 days after IVIg treatment) [25]. They compared it with the control group consisting of patients with infectious illnesses such as upper respiratory or gastrointestinal infections. The authors found that the sera from all KD patients stimulated NET formation during the acute phase. Notably, serum from severe cases of KD showed larger spider-like NET structures compared to milder cases. They also observed that neither the sera from the convalescent phase nor the control group induced NET formation. Dose-dependent inhibition of NET formation, oxidative burst, and decrease in neutrophil elastase was demonstrated by Masso-Silva et al. in patients with COVID-19 infection following treatment with IVIg [26]. Furthermore, Hu et al. also demonstrated reduced NET formation in patients with KD following treatment with IVIg [27]. These findings suggest that NETosis may play a role in the pathogenesis of KD, potentially contributing to the inflammatory response and vascular damage observed in the disease. IVIg treatment appears to have a suppressive effect on NET formation, which may contribute to its beneficial effects in managing KD. 

#### 3.1.2. Interaction with Macrophages (Figure 1)

IVIG had an inhibiting effect on FcγRs from monocyte-derived macrophages. Post IVIg infusion leads to down-regulation of FcγRIA and FcγR3A, thus repressing monocytes and monocyte-derived macrophages (MDMs). This is one of the plausible mechanisms of action of IVIG in KD. IVIg competes with immune complexes (ICs) for binding to the FcγRs, thereby preventing the activation of these cells by ICs [28]. Similar studies from Nagelkerke et al. have also found that blocking of formation of MDMs was most effective in the presence of immune complexes (dimeric and multimeric IgGs) [29].

#### 3.1.3. Interaction with Vascular Endothelial Cells (Figure 2)

Endothelial cells also function as sentinel innate immune cells. Low-density lipoproteins (LDL) and oxidized phospholipids can act as damage-associated molecular patterns (DAMPs), triggering inflammatory responses and altering the epigenetics of endothelial cells. This, in turn, potentiates inflammation by recruiting inflammatory cells such as neutrophils and monocytes and contributes to the pathogenesis of coronary arteritis in KD [30,31].

**Figure 2:**
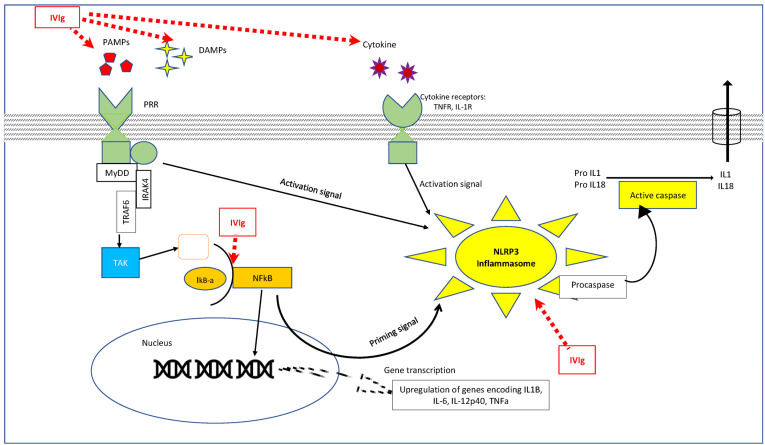
Immunomodulatory effect of IVIg on the humoral arm of the innate immune system and on inflammasome. NF-κB (Nuclear factor kappa B) signaling pathway is activated by the binding of ligands to PRR, TNFR, and IL-1R. Activation of this cascade leads to activation of kinase (IKK), which causes phosphorylation IkB-α, which releases NF-κB, which then translocates to the nucleus and regulates gene transcription of various cytokines, including NLRP3 (priming signal). The activation signal for inflammasome activation comes from either the binding of PAMPs/DAMPs onto PRRs or the damage signal from mitochondria. Upon activation, the NLRP3 complex assembles, causing the conversion of procaspase to active caspase and IL-1B and IL-18. IVIg inhibits the pathway at various steps, as indicated by dotted lines. PRR: patterned recognition receptor; TNFR: tumor necrosis factor receptor; IL-1R: interleukin-1 receptor; PAMPs: pathogen-associated molecular patterns; DAMPs: damage-associated molecular pattern; IVIg: intravenous immunoglobulin.

Hamamichi et al. reported that circulating neutrophils abundantly express vascular endothelial growth factor (VEGF) in the acute phase of KD, suggesting that neutrophil-derived VEGF may contribute to the vascular injury and remodeling observed in KD vasculitis [32]. Adhesion molecules such as CD11b, CD64, and CD62 have also been found to be upregulated during the acute phase of KD. These molecules are involved in the adhesion and migration of immune cells. Following IVIg therapy, the expression of these adhesion molecules has been observed to reduce, indicating the potential role of IVIg in modulating the inflammatory response and adhesion processes in KD [33,34].

#### 3.1.4. Interaction with NK Cells

Due to the ability of IVIg to inhibit the interaction between NK cells and endothelial cells, high-dose IVIg is supposed to be beneficial in patients with KD [13]. McAlpine et al. showed that administration of high-dose IVIg resulted in increased activity of NK cells and circulating CD16+ cells. In patients with KD post-IVIg treatment, T_reg_ cell frequency, activated CD25^+^ immunoregulatory CD56^bright^ NK cells, and expression of lymphoid tissue homing receptor (CD62L) were increased. Additionally, post-IVIg, there were reduced pro-inflammatory markers and decreased frequency of cytotoxic CD56^dim^ NK cells expressing the degranulation marker CD107a signifying a reduction in target cell killing. Another activating receptor, CD336, was expressed in a higher proportion on CD56^bright^ NK cells post IVIg in patients with KD, while other NK receptors were increased differentially. To conclude, IVIg treatment in patients with KD may have multiple effects on NK cell subsets, leading to the resolution of inflammation [35]. 

#### 3.1.5. Interaction with Dendritic Cells (DCs)

IVIg inhibits the differentiation and maturation of DCs in vitro and abrogates the capacity of mature DC to secrete interleukin (IL)-12 on activation while enhancing IL-10 production [36]. A study by Wang et al. shows that IVIg therapy restores the quantity and phenotype of circulating DCs and CD4^+^ T cells in children with acute KD exhibiting fewer peripheral DC subsets and CD4^+^ T cells. Authors have shown that in a study of 54 patients with KD, human leucocyte antigen-DR (HLA-DR) expression was reduced on CD1c^+^ myeloid DCs (CD1c^+^ mDCs), whereas that on plasmacytoid DCs (pDCs) did not change significantly. Both mDCs and pDCs showed significantly reduced expression of co-stimulatory molecules, including CD40 and CD86, along with the presentation of an immature or tolerant phenotype in the acute stages of KD. The phenotypes of DCs were found to be restored following IVIg treatment. The number of circulating pDC and CD1c^+^ mDC inversely correlated with plasma IL-6 levels in KD patients pre-IVIG treatment. However, no significant differences were found in patients with KD with and without CAAs [37,38]. 

#### 3.1.6. Interactions with Molecules in the Innate Immune System: (Figure 2)

PAMPs and DAMP: PAMPs are derived from molecular patterns conserved within a species of microbes and are recognized by patterned recognition receptors (PRRs) on certain innate immune cells. DAMPs are cell-derived molecules released in response to tissue damage, bind to PRRs, and induce innate immune response similar to PAMPs. Soluble innate pattern recognition molecules (PRMs) such as pentraxins, collectins (mannose-binding lectin), and M-ficolins are prime players of the humoral arm of innate immunity that can also activate complement pathways [39]. PAMPs have been shown to initiate coronary arteritis in mouse model study [40]. It has been shown that these DAMPs and PRMs are elevated in KD during the acute phase. Kusuda T et al. showed that certain PAMPS/DAMPs bind to IgG (Fc region) in in vitro experiments [41]. Thus, IVIg might exert its anti-inflammatory actions by adsorbing on PRRs and neutralizing them, preventing their action [42]. 

Signaling of IL-1 has also been found to be upregulated in patients with KD, suggesting the role of the inflammasome pathway in the pathogenesis of KD [43]. Recent studies have demonstrated the upregulation of inflammasomes, particularly NLRP3, in patients with KD [44,45]. IVIg has been shown to suppress NLRP3 and NLRP1 inflammasome-mediated cell death in other conditions, such as ischemic stroke [46]. This suggests a potential role of IVIg in modulating the inflammasome pathway in KD.

NF-κB, a transcription factor involved in the activation of proinflammatory cytokines and adhesion molecules, is also required for the activation of the inflammasome. Using in vitro experiments, Ichiyama et al. demonstrated that IVIg inhibits IκBα degradation, an inhibitor of NF-κB [47]. This inhibitory effect on NF-κB activation could contribute to the anti-inflammatory actions of IVIg. These findings highlight potential mechanisms through which IVIg exerts its anti-inflammatory effects in KD, such as neutralizing PRRs, modulating inflammasome activation, and inhibiting NF-κB signaling. However, further research is needed to fully understand the specific mechanisms involved and their relevance to the pathogenesis of KD.

### 3.2. Adaptive Immunity

Regulatory effects on B-lymphocytes and T-lymphocytes contribute to the immunomodulatory actions of IVIg in patients with KD.

#### 3.2.1. Interaction with T Cells

In vitro studies showed that IVIg suppresses T-cell proliferation and cytokine production while modulating T-cell differentiation. The suppression of Th17 differentiation from naïve human T cells and the blocking of Th17 cell proliferation and pro-inflammatory cytokine release was mediated by the Fab through inhibition of STAT3 phosphorylation. Increased HLA-DR expression in T cells is associated with IVIg resistance in KD patients [48]. Identification of immunodominant Fc epitopes capable of binding multiple HLA alleles could lead to the development of a valuable alternative to IVIg for KD patients.

Regulatory T cells (Tregs) play an important role in regulating the proinflammatory actions of effector T cells that take part in the destruction of the vessel wall. Studies have also shown that dysfunctional Treg may be associated with immune dysfunction in KD. Ni et al. showed that a decrease in FoxP3(+) Treg might be associated with decreased expression of micro-RNA [miR-155], leading to aberrant SOCS1/STAT-5 signaling and overexpression of miR-31 in patients with acute KD [49]. Natural Tregs have been involved in maintaining vascular homeostasis. Activation of natural Tregs recognizing the heavy constant region (Fc) of IgG is an important mechanism of action of IVIg therapy in KD. These results have been used in clinical trials using an Fc-enriched IVIg preparation, which showed similar efficacy to intact IVIg in patients with CAAs; IVIg enriched for Fab fragments was not effective in preventing CAAs, suggesting that beneficial effects of IVIG are mediated through Fc [50]. Lack of circulating Fc-specific natural Treg in the sub-acute phase of KD is correlated with the development of CAAs [51]. Fc-specific natural Treg was detectable in pediatric patients with various acute infections associated with fever and inflammation but not in acute KD patients prior to IVIg treatment. The decrease in CD25^+^CD4^+^ regulatory T cells during the acute phase might have a role in the development of KD. These cells have been attributed to maintaining immunologic self-tolerance and antimicrobial immune responses [52].

#### 3.2.2. Interaction with Antibodies

Several autoantibody responses have been reported in children with KD, and antibodies have been generated from aneurysmal plasma cell infiltrates [53]. IVIg can neutralize autoantibodies and inhibit the expansion of autoreactive B lymphocytes by inducing cell cycle arrest and B-cell apoptosis. This is one of the mechanisms which is proposed in patients with KD. However, there is no evidence of a direct pathogenic role for autoantibodies in the pathogenesis of KD. Additionally, IVIg has been suggested to delay the maturation of germinal centers, which are sites of B-cell proliferation and antibody production [54].

## 4. IVIg Refractory KD

Most children with KD respond to the initial dose of IVIg infusion. Those who develop a recrudescent or persistent fever at least 36 h after the end of their IVIg infusion are termed IVIg-resistant KD. The prevalence of IVIg-resistant KD is between 10 and 20% [55]. It constitutes a significant risk factor for the development of CAAs. These children require additional treatment with infliximab, corticosteroids, cyclosporine, or anakinra [55]. Genome-wide association studies (GWAS) studies have shown single nucleotide polymorphisms (SNPs) in genes of IFN-gamma, DC-SIGN, IL-1B, FcγR, MRP4, BAZ1A, STX1B, high mobility group box 1 (HMGB1), and P2Y12 (P2RY12) have been associated with risk of IVIg unresponsiveness [56,57,58,59,60,61]. Numerous scoring systems have been developed for the prediction of IVIg resistance. These include the Kobayashi score, Sano score, and Egami score [62,63,64]. However, these scoring systems have failed to show accurate predictions in other ethnic groups [65]. Nevertheless, age < 1 year, hypoalbuminemia, elevated transaminases, and neutrophilic leukocytosis have been consistently associated with a higher risk of IVIg resistance and the development of CAAs [55]. A meta-analysis by Li et al. has shown that the risk of IVIg resistance increases with elevated acute phase reactants (erythrocyte sedimentation rate, C-reactive protein, polymorphonuclear leucocytes), total bilirubin, transaminases, pro-Brain natriuretic peptide (pro-BNP), and lower platelet count, hemoglobin and serum sodium [66]. Patients with severe KD show lower levels of serum IgG, which may be related to IVIg resistance and increased incidence of CAAs [62,67]. Recently echocardiographic abnormalities such as coronary artery dilatation, perivascular brightness, presence of pericardial effusion, left ventricular (LV) insufficiency, and mitral insufficiency in the initial period of illness have been associated with a higher risk of IVIg resistance and CAAs [68,69,70,71,72] 

## 5. Time to IVIg Administration since Onset of Fever—Early versus Late IVIg Treatment

Timely administration of IVIg in patients with KD is crucial in reducing the risk of developing CAAs. CAAs can occur in approximately 25% of untreated cases of KD, but the risk is significantly reduced to <5% with timely treatment. While there are no specific guidelines on the earliest possible timing for IVIg, initiating treatment as early as possible is generally recommended, ideally within the first 10 days of fever onset. The rationale behind this recommendation is to coincide with the peak of systemic inflammation, typically observed between 5–10 days in most studies [55]. Recent AHA guidelines suggest that the diagnosis of KD can be made as early as 3 days of fever by an expert if typical features are present, highlighting the importance of early diagnosis and timely treatment [73]. Few studies have found an association between early administration of IVIg (as early as day 4 of illness) and a higher requirement of additional treatment as well as the risk of relapse of fever after initial resolution [74]. However, it is possible that patients who receive IVIg early in the course of illness may have a more severe disease with a higher degree of inflammation and typical clinical signs leading to the early identification and treatment of KD and that these subsets of patients may be already at a higher risk of developing CAA despite early treatment. Cai et al., in a retrospective analysis, compared different timing groups of IVIg treatment, i.e., early (<5 days), conventional (5–7, 7–9 days), and late groups (>/= 10 days), and found no difference in rates of IVIg resistance between the groups and authors observed an increased rate of CAAs in the late group in comparison to conventional group [75]. A recent meta-analysis by Yan et al. suggests that early IVIg treatment (</= 5 days) did not significantly reduce the incidence of CAAs overall. However, the analysis found regional differences in the outcomes—Japan showed no significant difference in CAAs development (OR 1.27; *p* = 0.074); however, studies from the United States and China showed a reduced risk in the occurrence of CAAs with early IVIg treatment (OR 0.73; *p* = 0.000 and OR 0.50; *p* = 0.000, respectively) [76]. These regional variations may be attributed to differences in patient populations, disease severity, or other unidentified factors. These findings highlight the complexity of determining the optimal timing of IVIg administration in KD. 

## 6. Data on the Duration of IVIg Infusion 

The advantages of shortened infusion duration are that it shortens the hospital stay, allows earlier determination of the efficacy of the initial IVIg, and allows to decide on additional treatment earlier. Additionally, shorter infusion periods may reduce the inflammation rapidly however is associated with the risk of headache, vomiting, and thrombosis due to rapid infusion rate [77]. AHA 2017 guidelines recommend administering 2 g/kg IVIg over 10–12 h [55]. A multicentric randomized trial by Fukui et al. assessed the safety and efficacy of the infusion rates (12 h vs. 24 h) in patients with KD during the acute phase and found no statistically significant difference in fever duration (21 h versus 21.5 h, *p* = 0.325), the requirement of additional IVIg (36.8% vs. 30%, *p* = 0.741) or a third line treatment (21.1% versus 5%; *p* = 0.182) or any difference in serious adverse effects between the two groups. The requirement for additional third-line treatment was associated with the risk score at presentation, not the infusion rate. Two patients in the 12-h group (one at presentation and another on day 7 of illness) had small coronary aneurysms that regressed in follow-up. However, none in 24 h group had CAAs. Though the serum IgG levels increased in both the treatment arms by day 2, the levels were lower in the former compared to the latter (2414 mg/dL vs. 3037 mg/dL, *p* =<0.01). It may be hypothesized that serum IgG levels may fall rapidly after the shorter infusion, and the systemic inflammation may remain unsettled, resulting in the need for additional treatment by itself. On the other hand, a slower elevation of serum IgG level by 24 h infusion may maintain a more prolonged anti-inflammatory effect. However, this study was confounded by the difference in risk score between the 2 groups (lower sodium and IgG values in the 12-h group) at the baseline, and thus the superiority of 12-h infusion could not be proven [78]. However, further trials with better study designs are required to conclude the effects of shorter infusions.

## 7. Effect of the Strength of IVIg Concentration—5% versus 10% 

A few studies have elucidated the efficacy and safety profile of 5% versus 10% IVIg concentrations for treatment in children with KD. Downie et al. showed that in Canadian children, a higher IVIg resistance rate was reported in patients who received 10% IVIg compared with those who received 5% IVIg. However, it was unclear whether the difference was solely due to the IVIg concentration or a change in the brand [79].

Oda et al. specifically compared the effectiveness of 5% and 10% IVIg from a single brand. The author reported that patients who received 10% IVIg had a shorter duration of infusion (half the infusion time than 5%) and fever compared to those who received 5% IVIg (10 vs. 13 h, *p* = 0.022). However, the two groups had no difference in adverse events, CAAs, or IVIg resistance. Among IVIg non-responders, the duration between initial IVIg and second-line treatment was significantly shorter in the 10% group (47 h vs. 49 h, *p* = 0.035), suggesting that early adjuvant therapy may help reduce CAAs [80]. A nationwide database from Japan compared the outcome between low and high concentrations of IVIg among 48,046 patients with KD and found that the resistance rate was higher in the 10% group compared to the 5% group (44.7% versus 21%, *p* = 0.008), but there were no statistically significant differences in duration of fever or CAAs noted between the two groups [81]. Similar findings were noted in another study by Han et al. [82]. To summarize, the choice of IVIg concentration may vary based on individual patient factors, local protocols, and the availability of specific brands. Further research and studies with standardized protocols are necessary to provide clearer recommendations on the optimal concentration of IVIg for KD treatment.

## 8. Recent Data on the Efficacy of Different Doses of IVIg in the Treatment of KD—1 g/kg versus 2 g/kg 

Following the landmark study in 1991, a single high dose IVIg at a dose of 2 g/kg became the standard of care for KD. Recent AHA guidelines recommend 2 g/kg during the acute phase of KD. However, it adds up to higher medical costs and a financial burden to the family. A few earlier studies have shown that medium dose IVIg was equally effective and was sufficient in the majority in preventing CAAs [4,6,83,84].

Moving towards cost-effective options, few recent studies have compared a single dose of medium and high doses of IVIg during the acute phase of illness. Lan He et al. conducted a randomized controlled study comparing three treatment regimens—i.e., 1 g/kg once, 1 g/kg on 2 consecutive days, and 2 g/kg once in the treatment of KD. This study found no significant differences in duration of fever, time to defervescence, length of hospital stay, acute phase reactants (white blood cells, C-reactive protein), platelet count, and hemoglobin at 72 h after completion of initial IVIg infusion. More importantly, the incidence of IVIg resistance and CAAs at 2 weeks (15.2%, 15%, and 12.9%, *p* = 0.837) and up to 6 months following illness was comparable between the two treatment groups [85]. Similar results were found in a national-wide database from Japan [86]. Matsuura et al., based on their study, have suggested a more stratified therapy according to risk scores where treatment was guided by Kobayashi risk score and less high-risk score [87]. In this study, patients in the high-risk group (Kobayashi score ≥ 5 points) received 2 g/kg IVIg and prednisolone, whereas the moderate-risk group (Kobayashi score < 5 points and less high-risk score ≥ 2 points) received 2 g/kg IVIg and those in the low-risk group (Kobayashi score < 5 points and less high-risk score < 2 points) received 1 g/kg IVIg treatment. The study found no significant differences between the groups in terms of treatment response (defined as afebrile within 24 h of initial IVIg) or the rate of CAAs (7.3, 3.8, and 2.3% of patients in the high-, moderate-, and low-risk groups, respectively, *p* = 0.26) [88]. It is important to note that individual patient factors, local protocols, and clinical judgment should be considered when determining the appropriate IVIg dose for each patient. Further research and studies with larger sample sizes and standardized protocols are necessary to confirm these findings and provide more definitive recommendations on using medium-dose IVIg in KD treatment.

## 9. Side Effect Profile

High-dose IVIg infusion in children with KD largely has a good safety profile. Common adverse effects observed during or following the IVIg infusion include infusion-related reactions—mild flushing, rash, urticaria, chills, nausea, vomiting, and hypotension—that are transient and mild and do not require treatment cessation. Rarely, there have been adverse events such as aseptic meningitis, renal impairment, thrombosis [77], worsening of congestive cardiac failure, neutropenia, splenomegaly [12], pericardial effusion [7], derangement in liver functions [10], and hemolytic anemia [89].

## 10. Conclusions

In conclusion, the research conducted on the use of IVIg in KD has significantly advanced our understanding of its pathogenesis in recent years. IVIg has long been regarded as the cornerstone of KD treatment and has demonstrated effectiveness in preventing CAAs. However, despite its established efficacy, the precise immunomodulatory mechanisms of IVIg in KD are still being actively investigated. While a single high dose of IVIg remains the standard of care, there is a growing exploration of various dosages based on risk scores to optimize treatment outcomes. Nevertheless, further research is necessary to evaluate the efficacy of different dosages and infusion rates of IVIg in preventing CAAs. Refining our understanding of the appropriate dosing regimens can enhance treatment strategies and improve outcomes in patients with KD.

## Data Availability

Not applicable.
